# The research on cycloastragenol in the treatment of brain metastases from lung cancer: mechanistic exploration of radiotherapy sensitization and amelioration of brain injury

**DOI:** 10.3389/fmed.2025.1616894

**Published:** 2025-07-04

**Authors:** Yanyan Tao, Jingwen Chang, Xinyi Zhu, Jingjing Han, Xinru Wang, Yun Sheng, Ziyi Sun, Fang Liu, Yu Tao, Hongyan Wu, Chen Yu, Hao Liu, Fangtian Fan

**Affiliations:** ^1^Department of Emergency Medicine, The First Affiliated Hospital of Bengbu Medical University, Bengbu, Anhui, China; ^2^School of Pharmacy, Bengbu Medical University, Bengbu, China; ^3^Anhui Engineering Technology Research Center of Biochemical Pharmaceutical, Bengbu, China; ^4^Institute of Biomedical Technology, Jiangsu Medical College, Yancheng, China; ^5^Department of Integrated TCM & Western Medicine, The Affiliated Cancer Hospital of Nanjing Medical University and Jiangsu Cancer Hospital and Jiangsu Institute of Cancer Research, Nanjing, China

**Keywords:** Cycloastragenol, neutrophil Infiltration, neuroinflammation, lung cancer brain metastases, radiation-induced brain injury

## Abstract

**Objective:**

This study aimed to investigate the radiosensitizing and toxicity-reducing effects of Cycloastragenol (CAG) in the radiotherapy of lung cancer brain metastases.

**Methods:**

A brain metastasis model of lung cancer was established using stereotactic brain localization. After successful modeling, varying doses of CAG (5 mg/kg, 10 mg/kg, 20 mg/kg) were administered via intraperitoneal injection to evaluate its antitumor efficacy. Radiotherapy (3 Gy per session, total 10 sessions) was combined with CAG (20 mg/kg) to assess its radiosensitizing effects. Small-animal *in vivo* imaging was employed to evaluate antitumor efficacy and radiosensitization. Cognitive changes in mice were assessed using the novel object recognition test and the cylinder test. Neuroinflammatory responses in brain tissues were detected via immunofluorescence and qPCR. Transcriptome sequencing and network pharmacology were utilized to identify potential targets and mechanisms, while molecular docking validated interactions between CAG and key targets. Both *in vitro* and *in vivo* studies were conducted to elucidate the mechanisms underlying CAG’s adjuvant effects in radiotherapy, including enhancing efficacy and mitigating toxicity.

**Results:**

1. CAG significantly suppressed the growth of Lewis lung carcinoma (LLC) brain xenografts. 2. CAG markedly enhanced the radiotherapeutic efficacy against lung cancer brain metastases. 3. CAG ameliorated radiation-induced brain injury in tumor-bearing mice by attenuating pro-inflammatory polarization of microglia/macrophages. 4. CAG inhibited the activity of the JAK/STAT signaling pathway in LLC brain tumor tissues, thereby downregulating the expression of neutrophil chemotaxis-associated cytokines, including CXCL3 and CCL5. 5. CAG alleviated radiation-induced brain injury in tumor-bearing mice by suppressing the IKK/NF-κB signaling pathway in LLC brain tumor tissues, which further modulated microglial/macrophage pro-inflammatory polarization.

**Conclusion:**

CAG ameliorates neuroinflammation, enhances the therapeutic efficacy of radiotherapy for lung cancer brain metastases, and mitigates radiation-induced brain tumor injury by suppressing the activity of the JAK/STAT and IKK/NF-κB signaling pathways within metastatic lesions.

## 1 Introduction

Cancer, a disease that remains challenging to overcome, poses a significant threat to global health. According to 2023 cancer statistics, lung cancer ranks second in incidence but first in mortality (1). Despite advancements in therapies such as radiotherapy, the prognosis for advanced lung cancer patients remains poor due to distant metastases, particularly brain metastases (2, 3). Lung cancer brain metastases not only impair patients’ quality of life but also severely limit treatment options. Epidemiological data indicate that brain metastases occur in approximately 20–40% of lung cancer patients (4, 5). Current treatments for brain metastases face limitations: the blood-brain barrier (BBB) restricts drug penetration into brain tissue, necessitating multimodal approaches. Surgical resection and radiotherapy are common interventions, but surgical resection is technically challenging, associated with low cure rates and high recurrence, while prolonged radiotherapy may induce radioresistance, significantly reducing therapeutic efficacy (6). Thus, current strategies for lung cancer brain metastases focus on symptom palliation, survival prolongation, and quality-of-life improvement (7).

Radiotherapy plays a pivotal role in treating lung cancer, including primary and metastatic lesions. It employs high-energy electromagnetic waves or particle beams to disrupt tumor cell DNA, inhibit cell division/proliferation, and suppress tumor growth (8). However, its efficacy is often limited by radioresistance and radiation-induced brain injury, which not only diminish therapeutic outcomes but also cause adverse effects such as cognitive impairment (9, 10). Existing studies report that neutrophil infiltration into tumors is a key mechanism underlying radioresistance (11, 12). Recent Science research reveals that radiotherapy exacerbates tumor hypoxia, chemotactically recruiting neutrophils to provide glycogen, promote tumor angiogenesis, and establish an immunosuppressive microenvironment, thereby driving radioresistance (13). Consequently, nhancing radiotherapy efficacy while mitigating radiation-induced brain injury remains an urgent research priority.

Over the past decades, Traditional Chinese Medicine (TCM) has attracted significant scientific interest for its adjunctive potential in augmenting conventional oncotherapies. In TCM, tumorigenesis is theorized to arise from deficiency of vital qi (zhengqi), qi stagnation and blood stasis, and phlegm-toxin accumulation. Consequently, TCM therapeutic principles emphasize tonifying vital qi, promoting blood circulation to resolve stasis, and clearing heat and toxins. The representative TCM formulation Shenqi Fuzheng Injection, composed of Astragalus membranaceus (Huangqi) and Codonopsis pilosula (Dangshen), is extensively utilized in clinical oncology. Its antitumor efficacy is primarily mediated through immunomodulatory mechanisms that enhance host immune responses (14–17). CAG, a hydrolysis product of astragaloside IV and a key active component of Astragalus and Shenqi Fuzheng Injection, has limited antitumor research. However, existing studies demonstrate that CAG enhances CD8^+^ T cell antitumor immunity by inhibiting MHC-I degradation, activates p53 to induce apoptosis in colon cancer cells, and promotes paclitaxel-induced apoptosis in gastric cancer cells via STAT3 activation (18–20).

**FIGURE 1:**
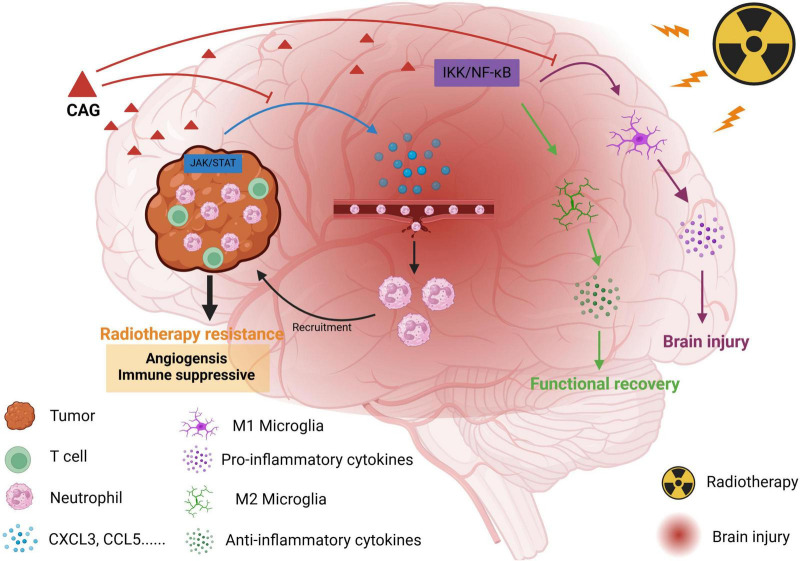
Schematic diagram of the effect of CAG on promoting radiotherapy against brain metastasis of lung cancer by inhibiting neutrophil infiltration.

This study initially examined the dose-dependent therapeutic impact of CAG - administered at low, intermediate, and high concentrations-on brain metastases originating from lung carcinoma. Subsequently, using a lung cancer brain metastasis model, we assessed its radiosensitizing effects and evaluated improvements in radiation-induced brain injury in tumor-bearing mice through behavioral experiments and qPCR analysis. Further, neutrophil-depleting antibodies are used to validate whether CAG exerts its radiosensitizing and neuroprotective effects by suppressing neutrophil infiltration. Finally, RNA-seq is employed to explore the mechanisms underlying CAG’s radiosensitization and neuroprotection, with validation via molecular docking, kinase activity assays, ELISA, and qPCR (Figure 1).

## 2 Materials and methods

### 2.1 Main reagents and consumables

(1) Cycloastragenol (source leaf), physiological sodium chloride solution (Fengyuan), T-25, T-75 sterile culture bottle, Trypsin (Biosharp), 1% streptomycin (Biosharp), 4% paraformaldehyde (Biosharp), 1 × PBS (Biosharp), DMEM medium (Gibco), 1,640 medium (Gibco), Fetal bovine serum (ExCell), Puromycin (Biosharp) pasteurized straw, 1 mL disposable sterile syringe, 10 mL round-bottomed capped centrifuge tube (Biosharp), 1.5 mL sharp-bottomed centrifuge tube (Biosharp), STAT5 inhibitor (STAT5-IN-1, HY-101853, MCE).

(2) Ki67 (Servicebio, GB111141), CD31 (Servicebio, GB11063-1), Ly6G (Servicebio, GB11229), Iba-1 (PTG, 26177-1-ap), CD16 (ABCam, ab183685), CD206 (CD206, ab182422) was used for IF.

(3) Mouse kits for ELISA: TGF-β1 (ruixinbio RX104768H), CXCL3 (ruixinbio RX101460H), CCL5 (ruixinbio RX105344H).

(4) PCR reagent: RNA extract (Xavier, G3013), Chloroform substitute (Xavier, G3014), Isopropanol (Xavier, 80109218), Reverse Transcription Kit (Xavier, G3337).

(5) Antibodies required for brain transplantation tumor experiment: InVivoMab anti-mouse Ly6G (BE0075-1. Bio X Cell).

(6) Reagents required for small animal *in vivo* imaging: D-fluorescein potassium salt (APExBIO), Isoflurane anesthetic (RWD).

### 2.2 Cell lines

In this study, we used the plasmid carrying puromycin resistance gene and luciferase reporter gene to construct the successful mouse lung cancer cell (LLC-Luc), which was purchased from Zhongqiao Xinzhou Company (LZQ0009). LLC-Luc cells were routinely cultured in a 5% CO_2_, 37°C incubator using high-glucose DMEM (Gibco) cell medium supplemented with 10% serum (FBS), 1% peniculin -streptomycin mixture (100X).

### 2.3 Mouse lung cancer brain transplantation tumor model and grouping

#### 2.3.1 Model making

To establish an intracranial tumor xenograft model, 6–8week-old male SPF C57BL/6J mice were used. The procedure was strictly conducted under sterile conditions. First, the mice were anesthetized using a small animal anesthesia machine and fixed on a stereotactic apparatus. The scalp of the mouse was incised from the ears to the eyes along the midline. Hydrogen peroxide was used to clean the skull and remove the fascia, exposing the cranial sutures. The bregma was located, and a hole was drilled in the skull at a position 1–1.5 mm below the coronal suture and 2–2.5 mm from the sagittal suture, using a mouse-specific skull drill. A 10-μL injection needle was used to draw up the LLC-LUC cell suspension, with a concentration of 1 × 10^5^ cells/μL and a total injection volume of 3 μL (3 × 10^5^ cells). The needle was carefully inserted to a depth of 3.5 mm below the skull, then retracted by 0.5 mm, and the cell suspension was injected. Finally, the skin wound was sealed with medical adhesive. The mice were placed on a heating pad until they recovered from anesthesia and were then returned to the animal facility for continued breeding.(21, 22).

#### 2.3.2 Grouping and drug administration

The tumor bioluminescence (BLI) signal was measured by the IVIS Xenogen imaging system 1 week after tumor cell injection. When the tumor bioluminescence signal reached 2 × 10^7^ photons/s (about 8 days later), the mice were divided into the following groups for subsequent experiments: model group, radiotherapy alone group (3 Gy), CAG group, radiotherapy combined with CAG group, radiotherapy combined with Anti-Ly6G (0.2 mg/mouse) group, and CAG combined with Anti-Ly6G group.

CAG was administered by intraperitoneal injection at doses of 5 mg/kg, 10 mg/kg, and 20 mg/kg. Anti-Ly6G was injected intraperitoneally at a dose of 0.2 mg per mouse.

#### 2.3.3 Small animal *in vivo* imaging techniques

The mice were intraperitoneally injected with fluorescein methyl ester (150 mg/kg) and anesthetized with a small animal anesthesia machine 10 min later. The mice were then placed into an IVIS Xenogen imaging system, and the bioluminescence imaging mode was selected.

### 2.4 Radiation therapy

The successful modeling mice were fixed on the Elekta Precise linear accelerator using isoflurane gas anesthesia. The radiation field was set to 40 cm × 1 cm, and the brain tumor was positioned within the radiation field. The dose rate and other parameters of the standard therapy mode built into the instrument were selected to follow the default settings, and the dose was set to 3 Gy. Radiation was delivered once a day for 10 days, starting when the intracranial fluorescence signal of the mice reached 2 × 10^7^ photons/s (21).

### 2.5 Immunofluorescence (IF)

Brain tissues were embedded in paraffin, subsequently sectioned using a microtome, and the wax sections were attached to polylysine-treated slides. The sections were dried at 60°C for 2–8 h, then deparaffinized with xylene I for 4 min and xylene II for 6 min. After rehydration with gradient alcohol for antigen retrieval, the sections were washed twice with PBS to remove residual xylene, and the PBS outside the specimen was blotted off with filter paper. Blocking serum was applied in a humidified chamber at 37°C for 30 min, followed by the primary antibody, which was incubated at 4°C overnight. The primary antibody was then removed by washing with PBS five times, and the PBS outside the specimen was blotted off with filter paper. The fluorescent secondary antibody was applied and incubated in a humidified chamber at room temperature in the dark for 1–1.5 h. Staining could be rapidly observed under a fluorescence microscope to determine the optimal termination time. The secondary antibody was removed by washing with PBS three times, and the PBS outside the specimen was blotted off with filter paper. The sections were then incubated with Hoechst 33,342 for 2 min in the dark to stain nuclei, which could be detected with blue fluorescence. Hoechst 33,342 was removed by washing with PBS three times, the PBS outside the specimen was blotted off with filter paper, and the sections were finally mounted with a mounting solution containing an anti-fade agent. The slides were immediately observed under a fluorescence microscope.

### 2.6 ELISA

The LLC cells treated with the drug were collected. The cells were lysed by repeated freezing and thawing. The lysate was centrifuged, and the supernatant was collected for detection.

For the experiment, the required salt was prepared, and standard wells, zero-value wells, blank wells, and sample wells were set up. Add 50 μL of different concentrations of standard solution to the standard wells. Add 50 μL of sample diluent to the zero-value wells. Do not add anything to the blank wells. Add 50 μL of the tested samples to the sample wells. In addition to the blank wells, add 100 μL of horseradish peroxidase (HRP)-labeled detection antibody to the standard wells, zero-value wells, and sample wells. Cover the reaction plate with a plate sealing membrane and incubate the plate in a 37°C water bath or incubator in the dark for 60 min.

After washing five times, mix substrates A and B thoroughly in a 1:1 volume ratio, and add 100 μL of the substrate mixture to all wells. Cover the reaction plate with a plate sealing membrane and incubate in a water bath or incubator at 37°C in the dark for 15 min. Then add 50 μL of stop solution to all wells and read the absorbance (OD) value of each well using a microplate reader.

Use the standard concentration as the abscissa (6 standard wells, plus 1 zero-value well, a total of 7 concentration points) and the corresponding absorbance (OD value) as the ordinate. Using computer software, fit a four-parameter logistic (4-PL) curve to create the standard curve equation. The concentration values of the samples were calculated using this equation.

### 2.7 qPCR

Take 5–20 mg of tissue and add 1 mL of RNA extraction buffer. Grind the mixture to homogenize it. Centrifuge the homogenate to remove the supernatant. Add 100 μL of chloroform, then centrifuge again to remove the supernatant. Add 550 μL of isopropanol and place the tube at −20°C for 15 min. Centrifuge at 4°C. The white precipitate at the bottom of the tube is RNA. Aspirate the liquid and mix the precipitate with 75% ethanol three times to wash it. Place the centrifuge tube on a laminar flow hood and dry for 3–5 min. Add 15 μL of RNA solubilization solution to dissolve the RNA. Measure the concentration and purity of the RNA using a NanoDrop 2000 spectrophotometer.

Reverse transcription was performed on a PCR apparatus using a reverse transcription kit (Xavier, G3337) according to the manufacturer’s instructions. A 0.1 mL PCR reaction plate was used to prepare the reaction system according to the instructions, with three replicates prepared for each reverse transcription product. After sampling, seal the plate with a PCR plate sealing membrane and a sealing instrument. Centrifuge the plate using a microwell plate centrifuge. Finally, perform PCR amplification on a real-time fluorescence quantitative PCR instrument (Table 1).

**TABLE 1:** Primers.

Name	Mouse source
TGF-β1	Forward:5′-AATGGTGGACCGCAACAACGCAATCT-3′
	Reverse:5′-TCTGGCACTGCTTCCCGAATGTCTGA-3′
CXCL3	Forward: 5′-GAATTCACCTCAAGAACATCCA-3′
	Reverse: 5′- AGTGTGGCTATGACTTCGG-3′
CCL5	Forward: 5′-ACCACTCCCTGCTGCTTT-3′
	Reverse: 5′-ACACTTGGCGGTTCCTTC-3′
GAPDH	Forward: 5′-AGGTCGGTGTGAACGGATTTG-3′
	Reverse: 5′-GGGGTCGTTGATGGCAACA-3′
CD16	Forward: 5′-GGAGTGATTTCTGACTGGCTGC-3′
	Reverse: 5′- GATGGAGGATGTAGTTGCTGGG-3′
INOS	Forward: 5′-AGCTCGGGTTGAAGTGGTATG-3′
	Reverse: 5′-CACAGCCACATTGATCTCCG-3′
CD206	Forward: 5′-GGAGTGGCAGGTGGCTTATG-3′
	Reverse: 5′-CACTGCTCGTAATCAGCCTCC-3′
IL10	Forward: 5′-GACTTTAAGGGTTACTTGGGTTGC-3′
	Reverse: 5′-CTTATTTTCACAGGGGAGAAATCG-3′
TGF-β	Forward: 5′-GCTGAACCAAGGAGACGGAATA-3′
	Reverse: 5′-GGCTGATCCCGTTGATTTCC-3′
IL17A	Forward: 5′-TCCACCGCAATGAAGACCCT-3′
	Reverse: 5′-CATGTGGTGGTCCAGCTTTCC-3′

### 2.8 New object recognition

Two identical objects were placed in a conventional open field box without a top. The mice were gently placed in the box for 10 min to familiarize themselves with the environment. After the mice were removed, they were placed in their home cages for 1 h. Then, one of the objects in the open field box was replaced with a different object, making the two objects distinct. The cognitive index of the mice was calculated using the following formula: Cognitive Index = (Exploration time of the novel object - Exploration time of the familiar object)/Total exploration time × 100%.

All behavioral experiments were conducted in a soundproof, temperature-controlled (25 ± 1°C), and uniformly illuminated (200 lux) environment and were assessed using a blind method, with all procedures performed by the same experimenter to minimize environmental interference and ensure that the operators were unaware of the grouping information.

### 2.9 Column experiment

The middle section of a 1.2-m-long stick was wrapped with emery cloth to increase friction. After positioning the stick horizontally, a mouse was placed at its center. Then, the stick was slowly lifted until it was perpendicular to the horizontal floor. Scoring was performed based on the following criteria (23) (Table 2).

**TABLE 2:** Scoring criteria.

Score	Time (s)
0	The stick fell before it was vertical
1	Less than 20s
2	20s–40s
3	40s–60s
4	More than 60s

### 2.10 RNA-seq

Total RNA was first extracted from LLC-transplanted tumors (RNA extraction was performed according to standard laboratory practices to ensure a high-quality RNA sample). Subsequently, the quality of the RNA extraction was assessed by testing the concentration and purity of the RNA. Next, the extracted RNA was treated with DNase I to remove any possible residual DNA. Subsequently, cDNA was synthesized by reverse transcribing the RNA using reverse transcriptase and random primers. After the completion of the cDNA synthesis reaction, a cDNA library was constructed for next-generation sequencing. In the process of library construction, steps such as end repair, adapter ligation, and library amplification are required. After library construction, the libraries were sequenced. During sequencing, platforms such as Illumina HiSeq or MiSeq are usually selected for high-throughput sequencing. After sequencing, raw data was obtained. The original sequencing data underwent quality control and preprocessing, including the removal of adapter sequences, low-quality sequences, and contamination, to ensure the accuracy of subsequent analysis. Subsequently, bioinformatics tools were used to align the cleaned data to the reference genome, calculate gene expression levels, and analyze differentially expressed genes. Finally, according to the experimental design and research purpose, further bioinformatics analyses such as functional enrichment analysis and pathway analysis can be performed to reveal the biological significance of changes in gene expression (21).

### 2.11 Molecular simulation

Firstly, three-dimensional structural data for STAT5B and Cycloastragenol must be acquired. The structure of STAT5B can be determined using experimental techniques such as X-ray crystallography and nuclear magnetic resonance (NMR), while the structure of Cycloastragenol can be sourced from database queries or computational chemistry methods. The structures of STAT5B and Cycloastragenol underwent preprocessing, which included operations such as the removal of water molecules, repair of structural defects, and the assignment of hydrogen atoms and charges, to ensure both structural integrity and reliability. The preprocessed structures of STAT5B and Cycloastragenol were then subjected to molecular docking using software such as AutoDock, DOCK, or Glide (among others) to perform molecular docking calculations. These software tools typically predict the binding sites and modes of interaction for STAT5B and Cycloastragenol based on the mechanical and energetic principles governing protein-small molecule interactions. The results from the docking calculations were used to assess the binding affinity, binding free energy, and potential binding sites of STAT5B and Cycloastragenol. To ensure the consistency of the results, multiple calculations were conducted during this process (21).

### 2.12 Statistical analysis

SPSS 25.0 was used to analyze the data, and the results were expressed as mean ± standard error of the mean (mean ± SEM). One-way analysis of variance (ANOVA) was used to compare differences among multiple groups, and Student’s *t*-test was used for comparisons between two groups. GraphPad Prism 7 was also used for statistical analysis. Statistical significance was determined as follows: ns (not significant) indicates *p* > 0.05, *p < 0.05, **p < 0.01, and ***p < 0.001.

## 3 Results

### 3.1 CAG inhibits the growth of LLC lung cancer brain transplantation tumors

**FIGURE 2:**
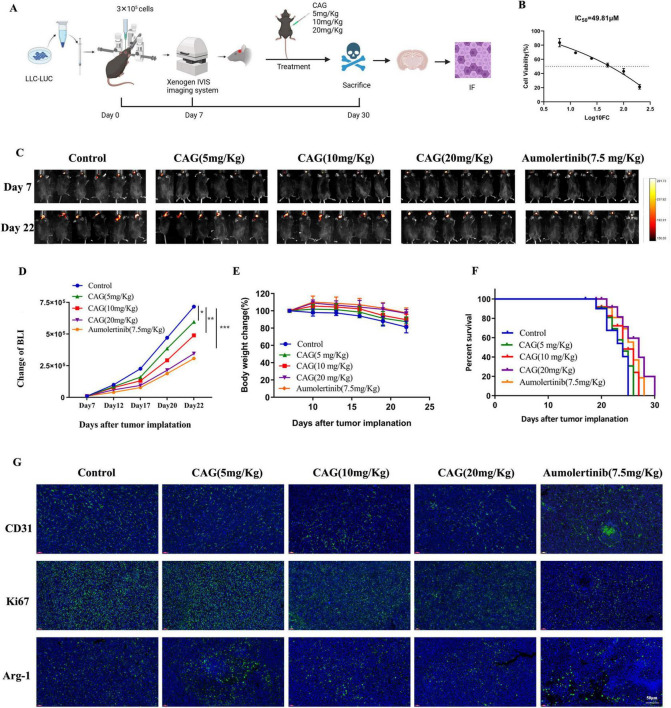
CAG inhibits the growth of LLC lung cancer brain metastasis tumor**s. (A)** Schematic diagram of the experiment. **(B)** Cytotoxicity of CAG on LLC cells. **(C)** Representative bioluminescence images of LLC brain tumor-bearing mice (*N* = 6 per group). **(D–F)** Changes in bioluminescence signal values, body weight, and survival rate during the treatment of LLC brain metastasis tumors (*N* = 6-8 in each group). **(G)** Immunofluorescence staining of tumor tissues in LLC-bearing nude mice under treatment, with indicators including CD31, Ki67, and Arg-1. (CD31 is used to label vascular endothelial cells, Ki67 is used to label proliferating cells, and Arg-1 is used to label M_2_-type macrophages.) Data are presented as mean ± SEM. *p < 0.05; **p < 0.01; ***p < 0.001.

To investigate the effect of CAG on the growth of brain metastases from lung cancer, LLC-Luc cells were used to establish a brain metastasis model in C57BL/6 mice. Mice with successfully established tumors were intraperitoneally injected daily with CAG at low (5 mg/kg), medium (10 mg/kg), and high (20 mg/kg) doses for the duration of the experiment. CCK-8 results indicated that the administered doses did not reach cytotoxic levels, and Almonertinib was used as a positive control (Figures 2A–C). The results demonstrated that CAG exhibited a dose-dependent inhibitory effect on LLC lung cancer brain transplant tumors. Specifically, a high dose (20 mg/kg) of CAG significantly improved the survival rate of tumor-bearing mice with minimal toxic side effects (Figures 2D–F). We further examined the expression of Ki67, CD31, and Arg-1 in LLC brain tumor tissues using immunofluorescence (IF). The results showed that a high dose (20 mg/kg) of CAG significantly suppressed the expression of CD31, Ki67, and Arg-1 (Figure 2G). These findings suggest that CAG effectively inhibits the growth of LLC-transplanted lung cancer brain tumors.

### 3.2 CAG enhances the effect of radiotherapy for brain metastases from lung cancer

**FIGURE 3:**
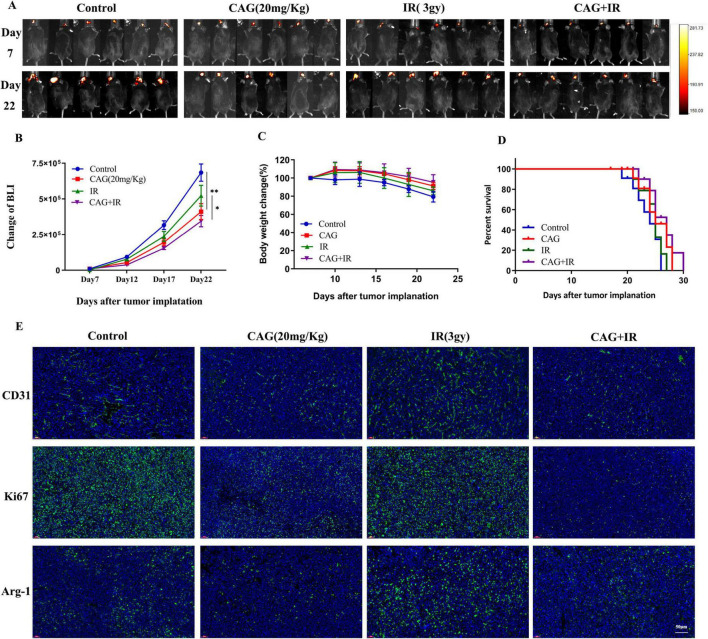
CAG enhances the effect of radiotherapy for brain metastases from lung cancer. **(A)** Representative bioluminescence images of LLC brain tumor-bearing mice. Mice were intraperitoneally injected with either normal saline or CAG (20 mg/kg) daily and irradiated with 3 Gy each time, for a total of 5 sessions (*N* = 6 per group). **(B–D)** Changes in bioluminescence signal values, body weight, and survival rate of LLC brain metastasis tumors during treatment (*N* = 6–8 in each group). **(E)** Immunofluorescence staining of tumor tissues in LLC-bearing nude mice under treatment, with indicators including CD31, Ki67, and Arg-1. Data are presented as mean ± SEM. *p < 0.05; **p < 0.01; ***p < 0.001.

To further explore the efficacy and mechanisms of CAG combined with radiotherapy, a lung cancer brain metastasis tumor model was established using LLC-Luc cells in C57BL/6 mice. After radiotherapy, which consisted of 5 sessions at a dose of 3 Gy per session, CAG (20 mg/kg) was administered intraperitoneally daily until the mice died. A series of results, including tumor bioluminescence signal values, body weight, and survival curves (Figures 3A–D), revealed that in the LLC brain metastasis tumor model, the CAG group significantly inhibited tumor growth compared to the control group. Compared with the CAG alone group and the radiotherapy alone group, the CAG combined with radiotherapy group exhibited a more significant inhibitory effect on tumor growth, which was essentially consistent with the efficacy of SFI. We used immunofluorescence (IF) to detect the levels of Ki67, CD31, and Arg-1 in LLC brain tumor tissues (Figure 3E). The results showed that compared with the control group, the CAG group decreased the expression levels of Ki67, CD31, and Arg-1. The levels of CD31 and Arg-1 were up-regulated in the radiotherapy group. Compared with the radiotherapy group, the expressions of CD31, Ki67, and Arg-1 were significantly down-regulated in the combined treatment group. This suggests that CAG enhances the effect of radiotherapy for brain metastases from lung cancer.

### 3.3 CAG alleviates radiation-induced brain injury in tumor-bearing mice by improving the pro-inflammatory polarization of microglia/macrophages

**FIGURE 4:**
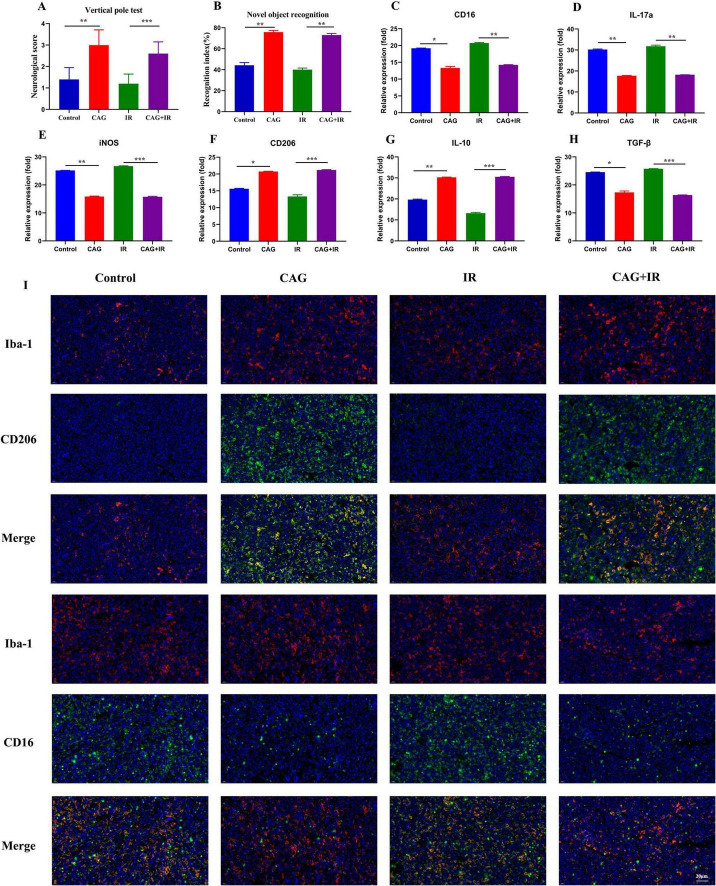
CAG ameliorates the pro-inflammatory polarization of microglia/macrophages to alleviate radiation-induced brain injury in tumor-bearing mice. **(A,B)** Behavioral scores of tumor-bearing mice in the novel object recognition and cylinder tests; **(C–H)** qPCR was used to analyze the expression levels of CD16, IL-17a, iNOS, CD206, IL-10, and TGF-β in LLC brain tumor tissues (*N* = 3). **(I)** Immunofluorescence staining of tumor tissues in LLC-bearing nude mice under treatment, with markers including CD206, CD16, and Iba-1. Data are presented as mean ± SEM. *p < 0.05; **p < 0.01; ***p < 0.001.

To further explore whether CAG can ameliorate radiation-induced brain injury in tumor-bearing mice, an LLC lung cancer brain metastasis tumor model was first established and divided into Control, CAG, IR, and CAG + IR groups. The results from the novel object recognition test and the cylinder test (Figures 4A, B) showed that CAG can enhance the learning, cognitive abilities, and neurological functions of tumor-bearing mice, and has a significant ameliorative effect on radiation-induced brain injury. It is well established that radiation-induced brain injury is closely related to neuroinflammation, particularly neuroinflammation mediated by microglial/macrophage polarization (24–27). We used qPCR to detect microglia/macrophage polarization markers in tumor-bearing brain tissue (Figures 4C–H), and the results indicated that the markers CD16 and IL-17a, associated with pro-inflammatory microglia/macrophages, were significantly reduced in the CAG group and the CAG combined with radiotherapy group. Concurrently, the expression of CD206 and IL-10, associated with anti-inflammatory microglia/macrophages, was significantly increased in the CAG group and the CAG combined with radiotherapy group. We further used immunofluorescence (IF) to double stain for CD16/Iba1 (pro-inflammatory) and CD206/Iba1 (anti-inflammatory), and the results (Figure 4I) demonstrated that CAG could inhibit pro-inflammatory microglia/macrophages and promote the polarization of anti-inflammatory microglia/macrophages following radiation-induced brain injury in tumor-bearing mice.

### 3.4 Network pharmacology was used to analyze the mechanism of CAG in enhancing the efficacy and attenuating toxicity of radiotherapy for lung cancer patients with brain metastases

**FIGURE 5:**
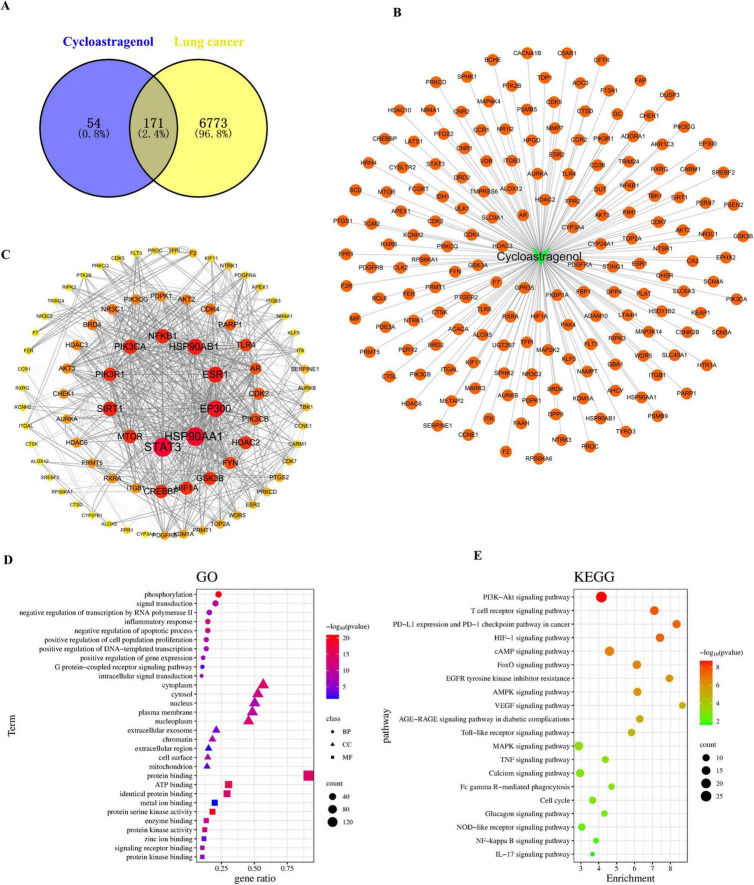
Network pharmacology was used to analyze the mechanism of Cycloastragenol in enhancing the efficacy and attenuating toxicity of radiotherapy for lung cancer patients with brain metastases. **(A)** The common targets between Cycloastragenol and lung cancer. **(B)** Drug-component-target prediction results. **(C)** Topological analysis of the protein-protein interaction (PPI) network. **(D)** Enrichment analyses of Gene Ontology (GO) terms. **(E)** Enrichment analyses of Kyoto Encyclopedia of Genes and Genomes (KEGG) terms.

The probability > 0 was used as the inclusion criterion for SwissTargetPrediction, and the targets of CAG were retrieved using SuperPred. 28 and 203 targets were included, respectively. After removing duplicates, a total of 230 component targets were obtained. A total of 6,474 lung cancer targets were included with a Relevance score > 5 as the inclusion criterion from the GeneCards database. 529 and 283 lung cancer targets were obtained through the OMIM and PharmGKB databases, respectively. The resulting genes were cross-referenced against the UniProt database. 171 intersection target genes of lung cancer and drugs were identified as the interaction target genes for drug treatment of lung cancer (Figure 5A). Using the drug-component-target data, Figure 5B was constructed, which includes 172 nodes and 171 edges. These 171 intersection target genes of lung cancer and drugs were identified as the interaction target genes for drugs in the treatment of lung cancer. The 171 intersection target genes obtained were imported into the STRING database^1^ for protein-protein interaction prediction, with the species set as Homo sapiens and the confidence level set as 0.7. The TSV file was saved in TSV format and imported into Cytoscape 3.8.2 software to draw the protein interaction network. Targets with a Degree value > 3 were selected, and the graph included 81 nodes and 774 edges. The network topology was analyzed; the degree value was used to reflect the size and color of the targets, and the combined score value was used to reflect the thickness of the edges, thus constructing the protein-protein interaction network, as shown in the figure. Among them, STAT3, HSP90AA1, EP300, HSP90AB1, ESR1, SIRT1, PIK3CA, NFKB1, MTOR, and PIK3R1 were the core targets (Figure 5C). Subsequently, GO and KEGG pathway analyses were performed to investigate and clarify the mechanisms of their predictions in depth. GO analysis showed that the following conditions were significantly enriched (Figure 5D): phosphorylation, inflammatory response, cytoplasm, protein binding, and signal receptor binding. KEGG enrichment results showed that these targets were mainly enriched in the PI3K-Akt signaling pathway, T-cell receptor signaling pathway, PD-L1 expression and PD-1 checkpoint expression in cancer, and the HIF-1 signaling pathway (Figure 5E).

### 3.5 Transcriptome analysis was used to analyze the mechanism of CAG in enhancing the efficacy and attenuating toxicity of radiotherapy for lung cancer patients with brain metastases

**FIGURE 6:**
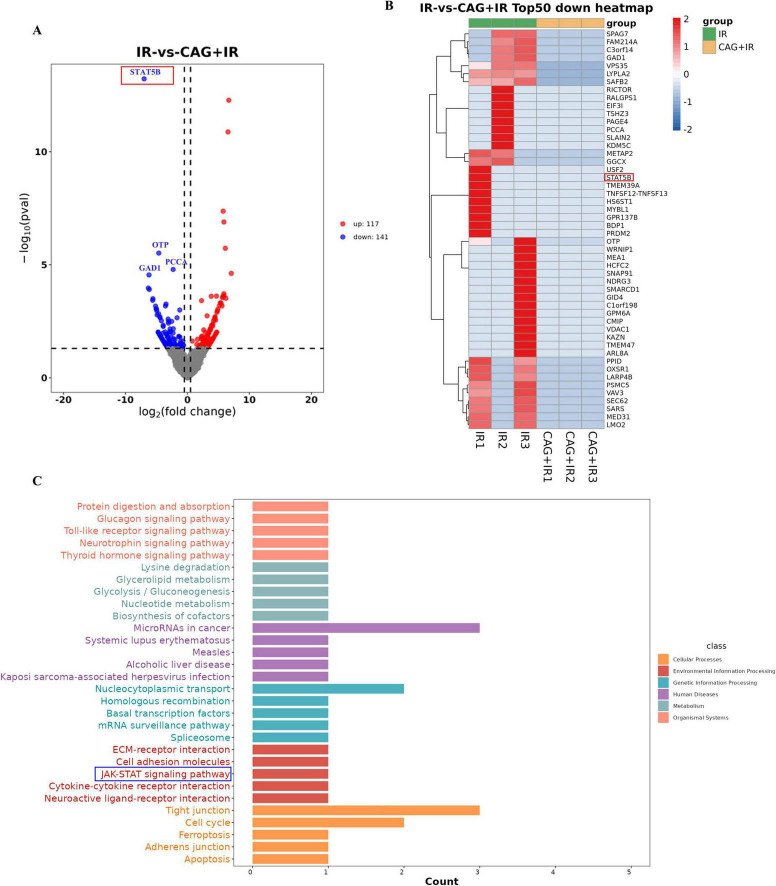
CAG significantly downregulated neutrophil chemokine-related signaling pathway activity in LLC brain tumor tissues after radiotherapy. **(A)** Differentially expressed genes between the radiotherapy group and the radiotherapy combined with Cycloastragenol group were summarized to form a volcano plot, with genes closer to the two ends being more differentially expressed. Red indicates up-regulated genes, and blue indicates down-regulated genes. **(B)** Differential genes between the groups were summarized, and the top 50 genes with the largest differences were used to form a cluster heat map, where red and blue represent high and low TPM (Transcripts Per Million) expression levels of the genes, respectively. **(C)** The KEGG enrichment analysis map was obtained through differential gene enrichment analysis, which was used to identify the significantly enriched signaling pathways among the differentially expressed genes.

To explore how CAG can enhance the efficacy and reduce the toxicities of radiotherapy for brain metastases of lung cancer, we performed RNA-seq analysis of brain tumor tissues from the radiotherapy group and the CAG combined with radiotherapy group. The volcano plot results indicated that CAG combined with radiotherapy had a lesser impact on gene transcription in LLC brain tumor cells *in vivo* RNA-seq experiments, with fewer gene differences observed. However, the STAT5B gene showed the most significant difference (Figure 6A). Statistical analysis of the top 50 differentially expressed genes revealed that the CAG combined with radiotherapy group down-regulated the expression of the STAT5B gene compared to the radiotherapy group alone (Figure 6B). KEGG enrichment analysis of the relevant differential genes from *in vivo* experiments indicated that CAG is closely related to the JAK/STAT signaling pathway (Figure 6C). Existing studies have shown that the JAK/STAT signaling pathway regulates the proliferation, survival, chemotaxis, and activation of neutrophils. This signaling pathway affects the function and chemotactic response of neutrophils by regulating chemokines such as CXCL3 and CCL5. These results suggest that CAG may inhibit the expression of neutrophil chemotactic-related cytokines by suppressing the activity of the JAK/STAT signaling pathway. Current research indicates that the activation of NF-κB promotes the pro-inflammatory polarization of microglia/macrophages (28–31). From this, we speculate that radiotherapy might promote the pro-inflammatory polarization of microglia/macrophages by activating the NF-κB signaling pathway, ultimately leading to brain injury.

### 3.6 Molecular docking verification

**FIGURE 7:**
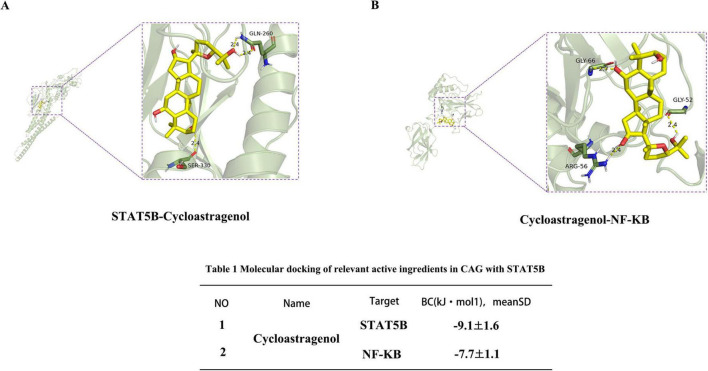
Interaction between Cycloastragenol and the STAT5B receptor. **(A,B)** Molecular simulation diagrams and interaction tables between Cycloastragenol and the STAT5B receptor.

To further verify the interactions between CAG and NF-κB as well as STAT5B, we conducted molecular docking simulations. After docking CAG with IKK/NF-κB and STAT5B, the binding energy of CAG with STAT5B was −9.1 kcal/mol, and that with IKK/NF-κB was −7.7 kcal/mol. This indicates that CAG has a strong binding affinity for both IKK/NF-κB and STAT5B (Figures 7A, B).

### 3.7 The inhibitory effects of CAG on NF-κB and STAT signaling pathways were verified *in vitro*

**FIGURE 8:**
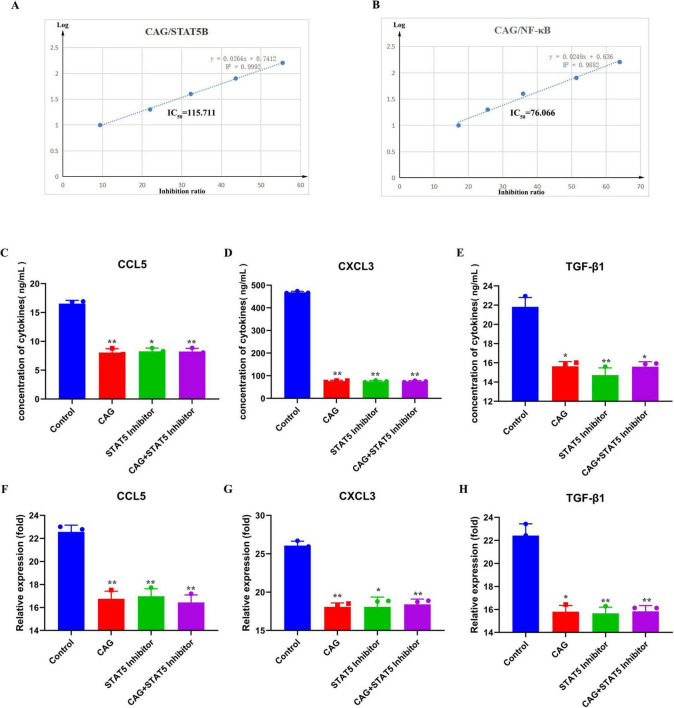
CAG inhibits neutrophil chemoattraction-related NF-κB and STAT signaling pathway activity. **(A,B)** Molecular simulation diagrams and interaction tables between CAG and the STAT5B receptor; **(C–E)** ELISA was used to analyze the effects of CAG and STAT5 inhibitor on the expression of TGF-β1, CXCL3, and CCL5 in LLC cells (N = 3). **(F–H)** qPCR analysis of the effects of CAG and STAT5 inhibitor on the expression of TGF-β1, CXCL3, and CCL5 in LLC cells (*N* = 3). Data are presented as mean ± SEM. *p < 0.05; **p < 0.01; ***p < 0.001.

To further confirm the effects of CAG on the expression of the NF-κB and STAT signaling pathways, *in vitro* kinase assays were conducted to verify the inhibitory effects of CAG on these pathways (Figures 8A, B). Subsequently, LLC cells were treated with a STAT5 inhibitor for ELISA and qPCR experiments (Figures 8C–H). The results indicated that the CAG group, the STAT5 inhibitor group, and the CAG combined with STAT5 inhibitor group all inhibited the expression of CCL5, CXCL3, and TGF-β1 compared to the Control group. Moreover, the levels of inhibition of CCL5, CXCL3, and TGF-β1 in the CAG group, the STAT5 inhibitor group, and the CAG combined with STAT5 inhibitor group were almost identical.

### 3.8 CAG enhances the radiotherapy effect of lung cancer brain transplantation tumor by inhibiting neutrophil infiltration.

**FIGURE 9:**
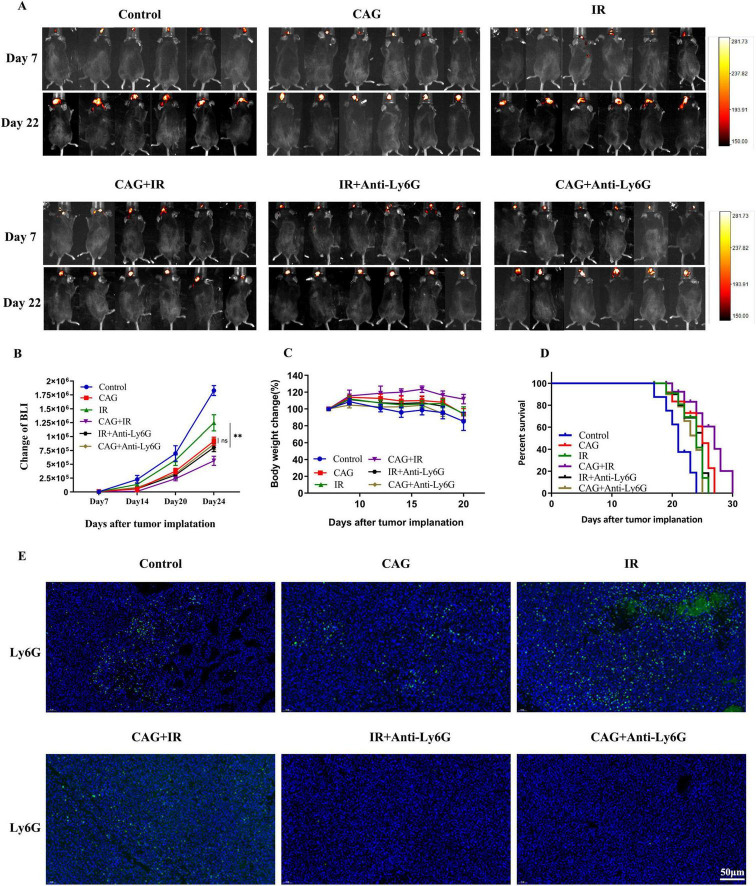
CAG enhances the effect of radiotherapy on lung cancer brain metastasis tumors by inhibiting neutrophil infiltration. **(A)** Representative bioluminescence images of LLC brain tumor-bearing mice; mice were intraperitoneally injected with either normal saline or Cycloastragenol (20 mg/kg) daily, and Anti-Ly6G was administered intraperitoneally every 3 days. The mice were irradiated with 3 Gy daily for 10 sessions (*N* = 6 per group). **(B–D)** Changes in bioluminescence signal values, body weight, and survival rate of LLC brain metastasis tumors during treatment (*N* = 6–8 in each group); **(E)** Immunofluorescence staining of tumor tissues in LLC-bearing nude mice under treatment, with Ly6G as the marker. Data are presented as mean ± SEM. *p < 0.05; **p < 0.01; ***p < 0.001.

In combination with mouse Anti-Ly6G, we investigated whether CAG exerts an anti-lung cancer brain metastasis effect by inhibiting neutrophil infiltration. In the LLC lung cancer brain metastasis tumor model constructed in C57BL/6 mice, we observed the tumor fluorescence signal value, body weight, and survival curve (Figures 9A–D): Compared with the Control group, CAG (20 mg/kg) significantly inhibited the growth of LLC lung cancer brain metastasis tumors. The effect of CAG combined with Anti-Ly6G was similar to that of CAG alone. Compared with the radiotherapy alone group, the combination of radiotherapy and Anti-Ly6G had a significant anti-tumor effect. The anti-tumor effect of CAG combined with radiotherapy was more significant than that of radiotherapy alone, which further confirmed that CAG enhanced the anti-brain metastasis effect of radiotherapy in lung cancer. We used IF to detect the level of Ly6G in LLC brain tumor tissues, and the results showed that the CAG group decreased the expression level of Ly6G compared with the control group. The expression level of Ly6G was up-regulated in the radiotherapy group. Compared with the radiotherapy group, the CAG combined with radiotherapy group significantly down-regulated the expression of Ly6G (Figures 9E). The above results confirmed that CAG inhibited neutrophil infiltration and played an anti-LLC lung cancer brain metastasis role.

### 3.9 CAG can alleviate radiation-induced brain injury by inhibiting the activity of NF-κB signaling pathway and improving the pro-inflammatory polarization of microglia/macrophages

**FIGURE 10:**
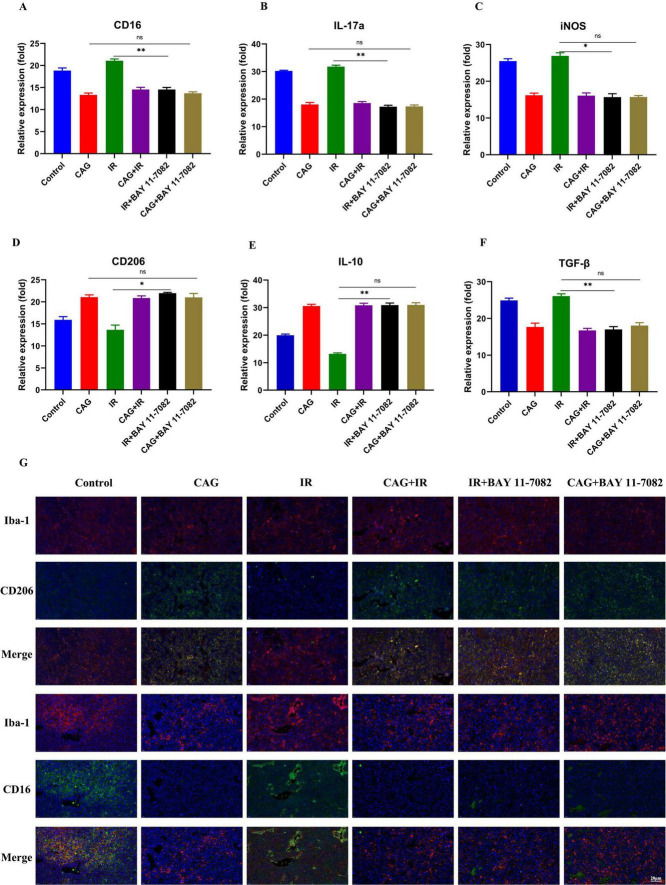
CAG alleviates radiation-induced brain injury by inhibiting the activity of the NF-κB signaling pathway and ameliorating the pro-inflammatory polarization of microglia/macrophages. **(A–F)** qPCR was used to analyze the expression of CD16, IL-17a, iNOS, CD206, IL-10, and TGF-β in LLC brain tumor tissues (*N* = 3). **(G)** Immunofluorescence staining of tumor tissues in LLC-bearing nude mice under treatment, with markers including CD206, CD16, and Iba-1. Data are presented as mean ± SEM. *p < 0.05; **p < 0.01; ***p < 0.001.

To investigate whether CAG could ameliorate the pro-inflammatory polarization of microglia/macrophages by inhibiting the activity of the NF-κB signaling pathway, we combined it with BAY 11-7082, an NF-κB inhibitor. LLC brain metastasis tumor models were established and divided into Control, CAG, IR, CAG + IR, CAG + BAY 11-7082, and IR + BAY 11-7,082 groups. BAY 11-7082 was administered intraperitoneally at a dose of 5 mg/kg twice a week. The expression of pro-inflammatory microglia/macrophage-related markers CD16 and IL-17a was significantly inhibited in the RT + BAY 11-7,082 group compared with the RT group (Figures 10A–F). Concurrently, it significantly enhanced the expression of anti-inflammatory microglia/macrophage markers CD206 and IL-10. The combination of CAG and BAY 11-7082, as well as CAG alone, showed comparable effects, inhibiting the expression of pro-inflammatory microglia/macrophage-related markers and promoting the expression of anti-inflammatory microglia/macrophage-related markers. The immunofluorescence (IF) results (Figures 10G) showed that, compared with the RT group, the combination of RT and BAY 11-7082 significantly inhibited the expression of CD16 and promoted the expression of CD206. The results of the CAG combined with BAY 11-7082 group were consistent with those of the CAG combined with radiotherapy group, and the results of the CAG group were also consistent with the CAG combined with BAY 11-7082 group. These results suggest that CAG can alleviate the pro-inflammatory polarization of microglia/macrophages by inhibiting the activity of the NF-κB signaling pathway.

## 4 Discussion

CAG is an active ingredient extracted from the traditional Chinese medicine Astragalus membranaceus. Studies have shown that CAG has a variety of biological activities, including antioxidant, anti-inflammatory, anti-cancer, and anti-aging effects (32). CAG also has anti-tumor effects, and existing studies have shown that CAG inhibits tumor development by inhibiting tumor cell proliferation and inducing apoptosis (18). In addition, CAG has been confirmed to have an immunomodulatory effect, which helps to enhance the body’s immune function (33). CAG plays a significant role in anti-aging. Studies have shown that CAG can prolong telomeres at the ends of chromosomes by activating telomerase, thereby slowing down the cellular aging process and improving the viability of cells (34). In this study, we investigated the effect of CAG on LLC brain metastases and enhancing radiosensitivity. Using Almonertinib as a positive control, high, medium, and low-dose CAG were given to LLC brain metastasis tumor mice, and we found that high-dose CAG had an anti-brain tumor effect. In this study, we further investigated the effect of CAG combined with radiotherapy on brain xenograft tumors in mice. We found that CAG had the effect of enhancing radiosensitivity and reversing radioresistance. According to existing reports, neutrophils promote tumor radioresistance; this study continued to investigate whether CAG enhances radiosensitivity by inhibiting neutrophil infiltration, and the results showed that CAG indeed enhances radiosensitivity by inhibiting neutrophil infiltration.

As an important part of the immune system, the study of neutrophils has been controversial (35, 36). Recent studies have found that neutrophils can promote the growth and spread of tumors by inducing inflammation and promoting angiogenesis in the late stages of tumors (37, 38). In the early stage, neutrophils play an anti-tumor role by inhibiting the immune escape mechanism of tumors and inhibiting tumor angiogenesis (39–41). Neutrophils can participate in the effect of tumor immunotherapy through a variety of mechanisms. Neutrophils can promote the presentation of tumor-associated antigens and the activation of T cells, thereby enhancing the anti-tumor effect of immunotherapy. In addition, neutrophils can also enhance the durability and effect of immunotherapy by inhibiting the activity of tumor-associated suppressor cells (42). Studies have found that neutrophils promote tumor immune escape and tolerance to radiotherapy and chemotherapy (11, 12). In this study, Anti-Ly6G was used to eliminate neutrophils in mice, and the results confirmed that CAG increased the radiosensitivity of LLC brain metastases in mice with high neutrophil infiltration.

Radiation-induced brain tumor injury is a serious complication. It can lead to cognitive decline, nervous system damage, and other neurobehavioral and emotional problems in patients (43). Firstly, radiotherapy can cause inflammation and oxidative stress in brain tissue. Radiation can induce the generation of free radicals inside and outside the cells, leading to increased oxidative stress, and then damage brain tissue (44). In addition, radiation can trigger the activation of inflammatory cells and the release of inflammatory factors, leading to the aggravation of the neuroinflammatory response and further damage to brain tissue. Secondly, radiotherapy may have adverse effects on the cerebrovascular system. The inflammatory response and oxidative stress caused by radiotherapy may lead to cerebrovascular endothelial injury and microvascular lesions, which in turn affect the blood supply and microcirculation function of the brain. This can lead to ischemia and hypoxia of brain tissue and aggravate the degree of radiation brain damage (24). Existing studies have reported that cordycepin provides long-term neuroprotection by inhibiting neutrophil infiltration and neuroinflammation after traumatic brain injury (TBI) (45). Based on this, this study investigated whether CAG could affect radiation-induced brain injury by interfering with neutrophil infiltration. The results showed that CAG could improve learning, cognitive ability, and neurological function and ameliorate radiation-induced brain injury in mice. By eliminating neutrophils in mice with Anti-Ly6G, we found that CAG enhanced the effect of radiotherapy on lung cancer brain metastasis tumors and alleviated radiation-induced brain injury by inhibiting neutrophil infiltration and improving neuroinflammation.

Based on the above experimental results, we found that CAG can inhibit the activity of the JAK/STAT signaling pathway in LLC brain tumors, and then inhibit the expression of neutrophil chemotactic-related cytokines such as CXCL3 and CCL5, and finally inhibit the infiltration of neutrophils to enhance the anti-tumor effect of radiotherapy and reduce the radiation damage of brain tumors (Figure 1).

In this study, a large number of animal experiments confirmed that CAG enhanced the effect of radiotherapy against brain metastasis tumors of lung cancer and reduced radiation damage of brain tumors by inhibiting neutrophil infiltration, and confirmed that CAG inhibited the expression of neutrophil chemotactic-related cytokines such as CXCL3 and CCL5 by inhibiting the activity of the JAK/STAT signaling pathway in LLC brain tumors. However, it was only verified by molecular simulation and molecular biology experiments, without further verification, and this study did not involve cell experiments for verification. Therefore, future studies need to further explore its internal mechanism to find new breakthroughs for the clinical treatment of lung cancer brain metastasis radiotherapy resistance and radiation brain injury.

## Data Availability

The datasets presented in this study can be found in online repositories. The names of the repository/repositories and accession number(s) can be found in the article/supplementary material.

## Footnotes

1
https://string-db.org/
